# RIVAWAR Trial Commentary: Rivaroxaban vs Warfarin in Acute Left Ventricular Thrombus Following Myocardial Infarction

**DOI:** 10.1016/j.jacadv.2025.102311

**Published:** 2025-11-12

**Authors:** Mahmoud Abdelnabi, Abdallah Almaghraby, Ramzi Ibrahim, Chadi Ayoub, Reza Arsanjani

**Affiliations:** a Department of Cardiovascular Disease, Mayo Clinic, Phoenix, Arizona, USA; b Cardiology Department, Ibrahim Bin Hamad Obaidallah Hospital, Ras Al Khaimah, UAE

We read with great interest the RIVAWAR (Rivaroxaban vs Warfarin in Acute Left Ventricular Thrombus Following Acute Myocardial Infarction) trial published by Shah et al.^1^ Although the investigators address an important clinical question by conducting one of the largest randomized trials to date, providing important data for anticoagulation with rivaroxaban vs warfarin for left ventricular thrombus (LVT) post-myocardial infarction (MI), we would like to highlight several concerns and discrepancies when compared with existing literature.^2^

First, the incidence of LVT implied by the trial’s enrollment appears unusually high. RIVAWAR, a single-center study enrolling 261 patients with LVT post-MI patients over ∼30 months. Even in high-risk MI populations in the primary percutaneous coronary intervention era, reported LVT incidence is estimated to be around ∼5 to 15%.^3^ The RIVAWAR recruitment rate suggests an unusually higher occurrence of acute LVT than previously observed, raising the possibility of overdiagnosis or uniquely high-risk cohort, which may limit generalizability. Clarification of the total MI population screened and diagnostic criteria used would help assess how representative this cohort is of typical post-MI cohorts.

Second, the trial applied 2:1 randomization in a single-center setting, unbalanced groups leading to potential bias. Even with baseline characteristics appearing grossly similar, the lack of stratified randomization may limit statistical power to detect differences in efficacy and safety endpoints. In addition, only per-protocol analysis was reported without intention to treat, which can compromise the balanced data created by randomization, introducing selection bias, risk of confounders, and ultimately overestimating treatment effects which was essential to maintain in this high-risk population.^4^

Third, given that LVT diagnosis and follow-up relied exclusively on transthoracic echocardiography (TTE) interpreted at the same center, without contrast use or blinded interpretation, the accuracy of LVT resolution assessment is uncertain. Noncontrast TTE has limited sensitivity, missing over 40% of LVT.^5^ Without confirmatory cardiac magnetic resonance or at least contrast-enhanced TTE, small persistent thrombi may have been overlooked, overestimating reported resolution rates. Furthermore, unblinded image interpretation may introduce potential detection bias. These imaging and blinding limitations should be acknowledged, as they impact the confidence in the reported outcomes. Finally, RIVAWAR trial reported unexpectedly high 3-months thrombus resolution in both arms (>95%), far exceeding those in prior studies, even with triple therapy (75% to 80%).^2^^,^^5^ Although the investigators justified these rates with early treatment initiation and a younger, revascularized population, the trial's short follow-up period and reliance on noncontrast TTE make the generalizability of the results questionable.

Despite these limitations, the RIVAWAR trial provides valuable insights, suggesting that rivaroxaban may be as effective as warfarin in post-MI LVT, with both achieving impressively high 3-month resolution rates. However, the unprecedented LVT resolution rates, combined with certain design constraints previously mentioned, and differences from prior studies, highlight the need for cautious interpretation. Independent, multicenter replication, using standardized imaging modalities such as contrast TTE or cardiac magnetic resonance with blinded assessment, extended follow-up, and intention-to-treat analysis will be important to more clearly define the optimal duration and approach to LVT treatment.
